# Surface-enhanced infrared absorption studies towards a new optical biosensor

**DOI:** 10.3762/bjnano.7.166

**Published:** 2016-11-16

**Authors:** Lothar Leidner, Julia Stäb, Jennifer T Adam, Günter Gauglitz

**Affiliations:** 1 Institute of Physical and Theoretical Chemistry (IPTC), Eberhard Karls University of Tübingen, Auf der Morgenstelle 18, 72076 Tübingen, Germany

**Keywords:** attenuated total reflectance Fourier transform infrared spectroscopy (ATR-FTIR), direct optical sensing, mid-infrared regime (MIR), reflectometric interference spectroscopy, surface-enhanced infrared absorption (SEIRA)

## Abstract

Reflectometric interference spectroscopy (RIfS), which is well-established in the visual regime, measures the optical thickness change of a sensitive layer caused, e.g., by binding an analyte. When operated in the mid-infrared range the sensor provides additional information via weak absorption spectra (fingerprints). The originally poor spectra are magnified by surface-enhanced infrared absorption (SEIRA). This is demonstrated using the broad complex fluid water band at 3300 cm^−1^, which is caused by superposition of symmetric, antisymmetric stretching vibration, and the first overtone of the bending vibration under the influence of H-bonds and Fermi resonance effect. The results are compared with a similar experiment performed with an ATR (attenuated total reflectance) set-up.

## Introduction

Optical biosensors play an important role in the detection and quantification of analytes. Among others, some applications include point-of-care testing (POCT), the monitoring of blood, urine, sudor and respiratory air, and the search for metabolites and markers for many diseases. Optical biosensors are used in basic research and life science, for example, to study protein–protein interactions [1–2]. A subfield of optical biosensors are those operated through direct optical detection. Direct optical sensing relates to the detection of analyte molecules without the use of labels. They lack the disadvantages of fluorescence technologies such as photodegradation, loss of bioactivity or costs of labeling. An early overview in the field of direct optical sensors, including optical principles and assay formats for selective detection, is given in [3] and updated in [4].

Besides sensitivity, stability, and reversibility, selectivity is a vital requirement of a biosensor, which is the main issue of direct optical method: it is not selective as a matter of principle. Optical sensors monitor the interaction of electromagnetic radiation with matter. In many optical sensors, if not most cases, a change of the refractive index is observed. In order to be selective to the analyte, but insensitive to unspecific binding, the sensor surface is covered by a biolayer which must guarantee both selectivity and sensitivity for the analyte under consideration.

There are many optical biosensors operating in the visible or near-infrared wavelength regimes with great success. Shifting the detection window to the mid-infrared (MIR) region has some advantages, but also creates many new problems to be solved. In his review article, Mizaikoff [5] asks the rhetorical question (and gives a positive answer of course): “Do we need to advance into the MIR spectral regime given the maturity of optoelectronics and photonics in the VIS and NIR?” The main reasons to research sensors that operate in the mid-infrared are the inherent molecular selectivity (“fingerprint spectra”), larger sensing volume (for evanescent field sensors due to the enhanced penetration depth), and the possibility to extract additional information by chemometric methods. An additional advantage is the increasing manufacturing tolerance compared to the visible region.

This leads to the proposal to combine different measurement techniques in order to gain more information. One idea is to operate a Mach–Zehnder interferometer (MZI) in the mid-infrared, thus combining the interferometric information (quantification) with the characteristic fingerprint (identification) of the attached analyte [6].

An example of such a combination (reported previously) is a gas sensor, which combines RIfS and MIR fingerprints as sensing principles. Gas molecules diffuse into a polymer film and cause a thickness change of the film. The thin film interference spectrum is used for determination of gas concentration. Identification of the analyte type was obtained from the mid-infrared part of the spectrum [7].

Using fingerprint spectra extracted from biosensor signals for the identification of analytes is therefore promising. However, in many cases the absorption signal is weak. A sensor whose sensing volume is based on the evanescent field of a guided wave (as is the above-mentioned MZI sensor), will show only a moderate absorption spectra, as the sensing volume is limited by the penetration depth of the evanescent field. This fact is both an advantage and a disadvantage. Biosensors often operate in aqueous solution, which strongly absorbs in the MIR. The small penetration depth ensures that the analyte spectrum is not obscured by the water bands. For the same reason, the analyte spectrum is poor. A solution to the problem of weak signal could be the implementation of surface-enhanced infrared absorption (SEIRA), which has gained some importance in the field of sensor applications during the last three decades [8–10]. The theory of SEIRA is similar to the theory of surface-enhanced Raman spectroscopy (SERS) where electrochemical and chemical mechanisms seem to be responsible for the signal enhancement [11]. The effect is caused by very thin metal films evaporated on a substrate, which consist of isolated islands with dimensions much smaller than the wavelength of light. According to Osawa, the island nature of the metal films is essential for the SEIRA effect. Therefore, it seems obvious to study SEIRA using metal nanoparticles. According to Yang and Griffiths [12], the ideal substrate for SEIRA is a thin film of isolated nanoparticles distributed over a suitable infrared transparent substrate. They report enhancement factors of approximately 100 for silver nanoparticles on a germanium substrate.

An interesting approach to signal enhancement is demonstrated by the surface-enhanced infrared attenuated infrared total reflection (SEIRA-ATR) set-up used by López-Lorente and co-workers [13]. Gold nanoparticles were directly synthesized within a liquid cell of an ATR unit and deposited on the surface of the ATR waveguide. The deposition of the water molecules leads to an increase of water absorption features due to adsorption of water molecules to the nanoparticle layer on top of the ATR waveguide.

In the current work, water absorption spectra have been studied under the influence of silver nanoparticles present in an aqueous solution by a RIfS set-up operating in the mid-infrared. Unlike ATR, where the monitored absorption occurs within the evanescent field of a guided, totally reflected wave mode, the RIfS signal is governed by the effects of reflection, transmission and absorption including interference caused by thin films.

## Results and Discussion

### Reflectometric interference spectroscopy in the mid-infrared

Reflectometric interference spectroscopy (RIfS) is a well-established sensor concept for direct optical sensing in the visible wavelength region of the electromagnetic spectrum. It can be used both as a chemical and a biological sensor by measuring the optical thickness change of a sensitive layer. The idea behind this concept is monitoring thin film growth by evaluation of the corresponding interference spectrum of a white light source. When the film is illuminated by a white light source, assuming perpendicular incidence, the reflectance *R* at the interfaces defined by the film and the surrounding media (substrate and superstrate) can be calculated according to


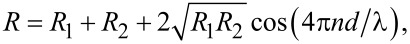


where *R*_1_, *R*_2_ are the Fresnel reflectances at the two interfaces, *d* is the physical thickness, and *n* the refractive index of the film [14].

Moving to the mid-infrared regime, the sensitivity to changes in film thickness is reduced compared to the visible wavelength regime, as the mid-IR wavelength regime is larger by a factor of 12–35 as compared to the visible region. Therefore, IR is less suited for detection and quantification of small molecules. However, it may be appropriate for detection of larger structures up to bacteria.

In this work emphasis is placed not on film thickness changes but on the accompanying absorption in the MIR, which in principle, can be used for identification of an analyte by its fingerprint.

### Measurements

Reflectance measurements of the RIfS set-up show an interference pattern caused by a polymer film on top of the GaAs substrate. Model calculations were performed in order to avoid coincidence between a minimum of reflectance and the spectroscopically interesting region between 3000 and 3400 cm^−1^ (data not shown here). Both a sensor with a Teflon layer (refractive index 1.29, layer thickness 1.65 µm) and a polycarbonate (PC) layer (refractive index 1.54, layer thickness 0.78 µm) were used in the experiments.

The measurements were performed in three steps. At first, the cell on top of the polymer layer was filled with double distilled water. Radiation transmitting the polymer will be nearly totally absorbed in water. The detector signal is composed of the interference of the reflected radiation at the air–GaAs, GaAs–polymer and polymer–water interfaces. In the absence of scattering nanoparticles, the intensity scattered from the water cover into the direction of the sensor will be negligible. As a consequence, there is only a negligible contribution of water absorption in the reflected signal.

In a next step an aqueous solution of 50 nm, stabilized, Ag nanoparticles is added to the water. A portion of nanoparticles deposit onto the polymer causing backscattering. There is nonzero transmittance from medium 1 (polymer film) to medium 2 (water). The transmitted light absorbs, interacts with the nanoparticles and is partially backscattered. As there is a wavelength dependent absorption, there is also a wavelength-dependent backscattering, part of which is directed to the detector. The spherical nanoparticles are responsible for a relatively weak water band.

When anisotropy is added to a nanoparticle, the optical properties change dramatically [15]. Therefore, as a last step of the experiment anisotropy is added by aggregation of nanoparticles. Addition of concentrated phosphate buffered saline (PBS, 10× concentrate) to the water–nanoparticles system causes the aggregation due to the salt effect of PBS.

**Figure 1:**
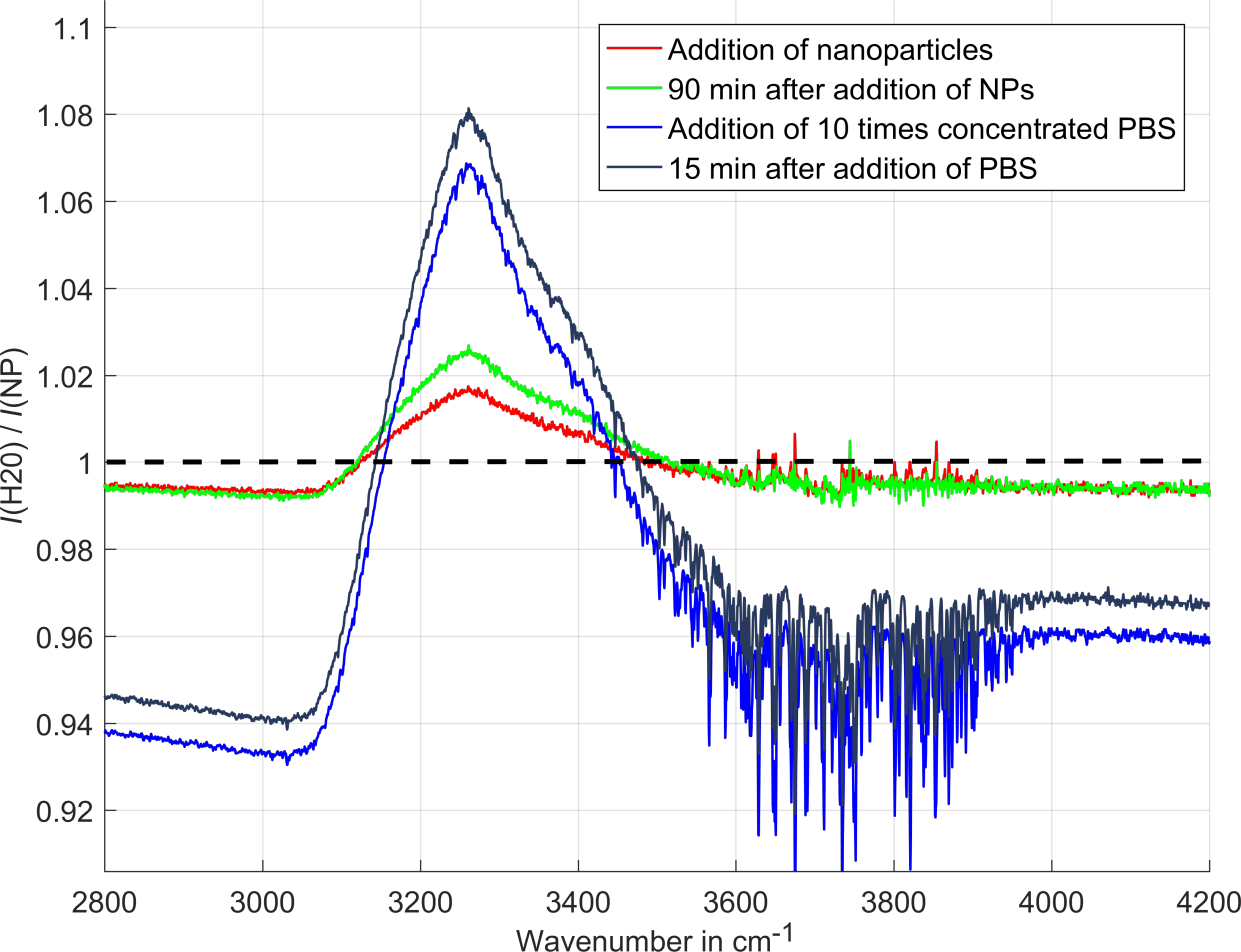
Amplification of water OH-stretching band (measurements with the Teflon sensor).

Figure 1 shows the results obtained with the Teflon AF2400 sensor (GaAs etalon, 6 mm thickness, coated by 1.65 µm Teflon AF2400). The quotient of the reflectance of water without nanoparticles (*I*_0_) and in the presence of nanoparticles (*I*) is plotted against the wavenumber.

The dashed line indicates the normalized absorption spectrum of double distilled water (*I*_0_/*I*_0_ = 1). After addition of an aqueous solution of 50 nm silver nanoparticles, the typical water features appear (a combination of water stretching and bending overtone bands). The signal increases and after about 90 min reaches its equilibrium (green line). 24 hours later, equilibrium is still preserved. After the addition of 10× concentrated PBS, there is a further increase of the absorption band. Equilibrium is reached 15 min after PBS is introduced.

The experiment was performed under ambient conditions (room temperature, optical path through humid air, relative humidity was not recorded). Air humidity is responsible for the sharp peaks between 3500 and 3800 cm^−1^ (rotation–vibration spectra of water vapor in air).

There is an obvious baseline shift which is small for nanoparticles and strong after aggregation. The reason could be that the aggregated particles are larger and therefore scatter a larger amount of light into the direction of the detector causing a nearly wavelength-independent baseline shift.

**Figure 2:**
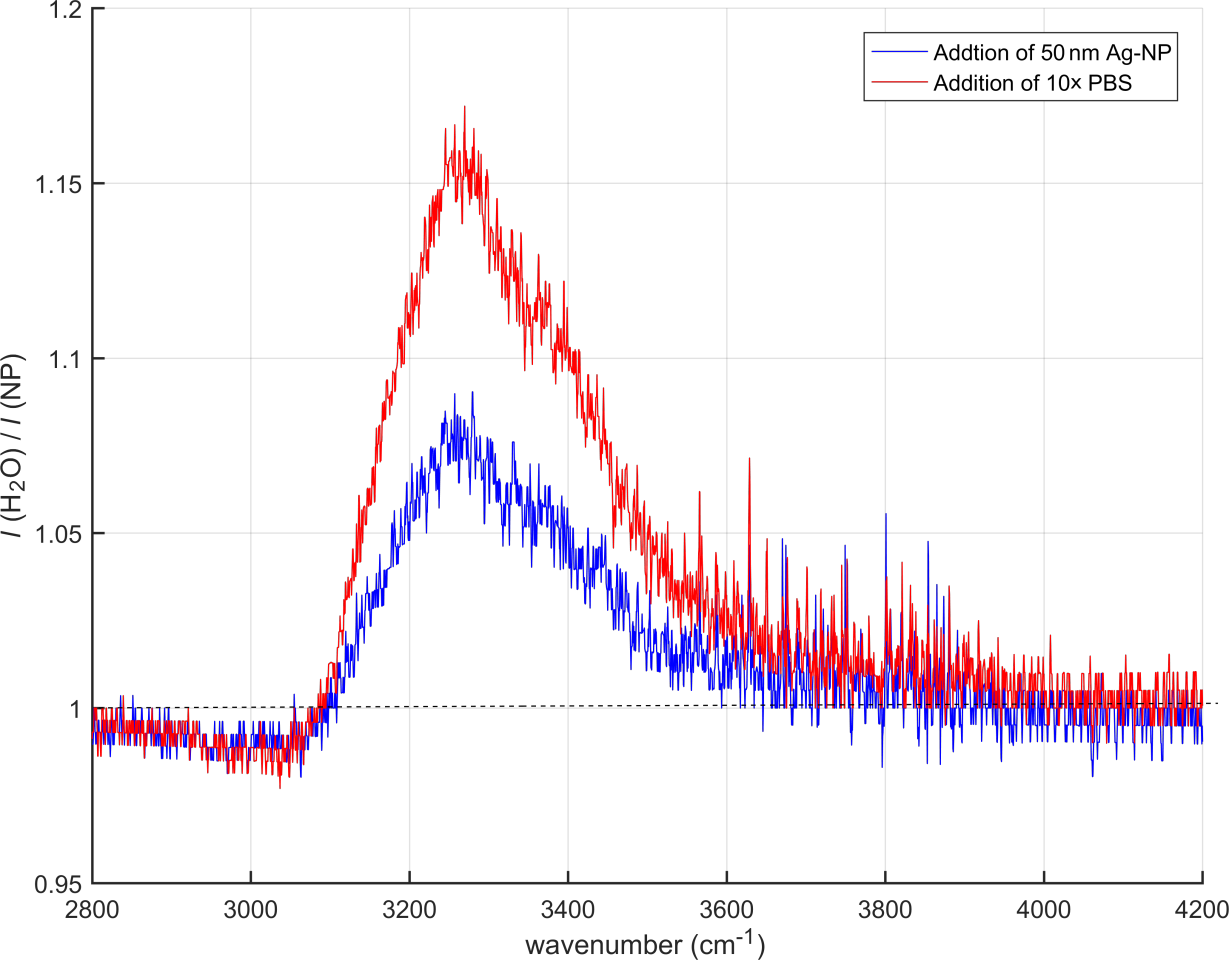
Amplification of water OH-stretching band (measurements with the polycarbonate sensor).

Figure 2 shows the results of a sensor chip with polycarbonate (PC) coating. This case shows the same finding: appearance of the water band by addition of nanoparticles, and further absorbance increase by PBS-induced nanoparticle aggregation. The band increase here is smaller than in the case of the Teflon sensor. A possible explanation is that the nanoparticle solution has aged, and aggregation may have already started before PBS addition.

## Discussion

Although the RIfS spectra look similar to the ATR spectra in [13], their origin is different. The ATR spectra are caused by absorption in the zone of the evanescent field above the ATR waveguide. In the case of the RIfS sensor, the interesting region is around the polymer–water interface. According to the Fresnel equations, a part of the radiation is reflected, the rest is transmitted into the aqueous solution above the polymer with its known strong absorption in the MIR. As the nanoparticles have a diameter of 50 nm (which is small compared to the wavelength in the MIR) they obey the rules of Rayleigh scattering. Therefore, nanoparticles, which have been deposited on the polymer backscatter a part of the transmitted radiation into the direction of the detector. Backscattered radiation is modified by the water molecules adsorbed to the nanoparticles and therefore subjected to SEIRA. So in the case of ATR, there is a sensitive zone limited by the penetration depth of the evanescent field, while for RIfS, the zone is limited by the strong water absorption. In both cases, the detected absorption peak has its maximum as soon as the sedimentation into the sensitive zone is finished.

As the reference (*I*_0_) is recorded with distilled water, there is no backscattering due to silver nanoparticles. As soon as nanoparticles are present, backscattering is a function of particle cross section. Aggregated particles have larger cross sections than nonaggregated particles. This is the proposed explanation of the baseline shift, which increases with increasing particle size in the *I*_0_/*I* spectrum.

The increase of the water absorption band in the case of aggregated nanoparticles may have two explanations: first, the SEIRA effect is enhanced when nanoparticles aggregate (addition of anisotropy, see [15]); second, the backscattering cross section is increased.

### Peak shift

**Figure 3:**
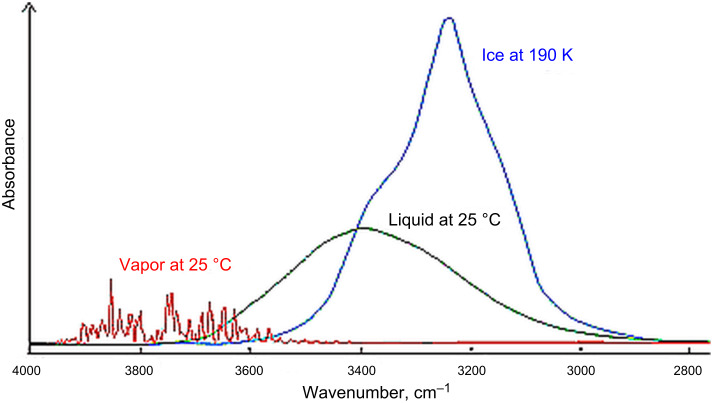
Comparison of the gas, liquid and solid spectra of the same amount of water, reproduced from [16].

A further interesting finding is the shift of the water absorption peak maximum in the presence of Ag nanoparticles in comparison to spectra of liquid water. In our measurements we have a peak maximum at about 3420 cm^−1^ for pure water in the absence of nanoparticles, which is in good agreement with published values. After addition of Ag nanoparticles, there is a shift of the absorption maximum to 3260 cm^−1^. The absorption features converge to the absorption spectra of ice (see Figure 3).

Looking at the spectra of Mizaikoff et al. [13], there seems to be a similar shift of the vibration spectrum to lower wavenumbers. However, no value of the peak maximum is given and the peak shift is not addressed in the text.

The discussions about the basic model of liquid water are still controversial [17–19], and will not be repeated here. However, the observed spectra suggest that there is an interaction between metallic nanoparticles and water structures.

In this context, the work of Ishida and Griffiths [20] is interesting. The authors investigated water bands by a germanium internal reflection element (IRE) with deposited copper films. They observed not only enhanced absorption (SEIRA) of the bending mode of water molecules in the presence of nanoparticulate copper particles, but also a band shift from 1640 to 1650 cm^−1^. The wavenumber increase of the bending mode and wavenumber decrease of the stretching mode seem to confirm the interaction between metallic nanoparticles and water structures.

## Conclusion

RIfS is a well-established sensor system working with a white light source in the visible part of the electromagnetic spectrum. Selectivity is gained by bio-functionalization of the sensitive interference layer on top of the layer system. This work investigated whether is it advantageous to migrate the spectrum to the mid-infrared regime and thereby gain enhanced selectivity by evaluation of the inherent molecular fingerprint. It was also examined whether the the fingerprint signal could further be amplified by the SEIRA effect.

Instead of constructing a new RIfS device with sensitive nanoparticles, their agglomerates were used to answer these questions. The work presented here is a pilot study with qualitative measurements only. Future experiments must be performed with quantitative input and a variety of analytes. Nevertheless, the results show evidence that there is an enhancement effect, even though small. But this is not the end of the story. The SEIRA effect can be enhanced by using specifically tailored nano-antennas, where enhancement factors up to 10,000 are reported [21]. Optimized nano-antennas with an optimized SEIRA effect, bio-functionalization of these antennas to avoid unspecific binding, and monitoring of film thickness growth by RIfS interference can be combined as a potential means to identify and quantify large molecules or even bacteria in an aqueous system.

As already mentioned in the Introduction, other optical sensors that successfully operate in the visible region of the electromagnetic spectrum could also benefit from migration to the mid-infrared spectrum. In a feature article Sieger and Mizaikoff [22], the potential for chemical and biological label-free sensing applications is emphasized. A toolbox containing tunable quantum cascade lasers (QCLs) and interband cascade lasers (ICLs) as promising radiation sources, as well as future quantum cascade detectors (QCDs), new mid-infrared waveguides together with nanostructured surfaces utilizing the SEIRA effect establish a promising field of research in mid-infrared label-free sensing.

## Experimental

### RIfS set-up

The mid-infrared spectra were recorded using a Bruker Equinox 55 FTIR spectrometer with a liquid nitrogen cooled MCT detector operated with OPUS 6.5 software. The RIfS setup is placed into the sample compartment of the spectrometer.

**Figure 4:**
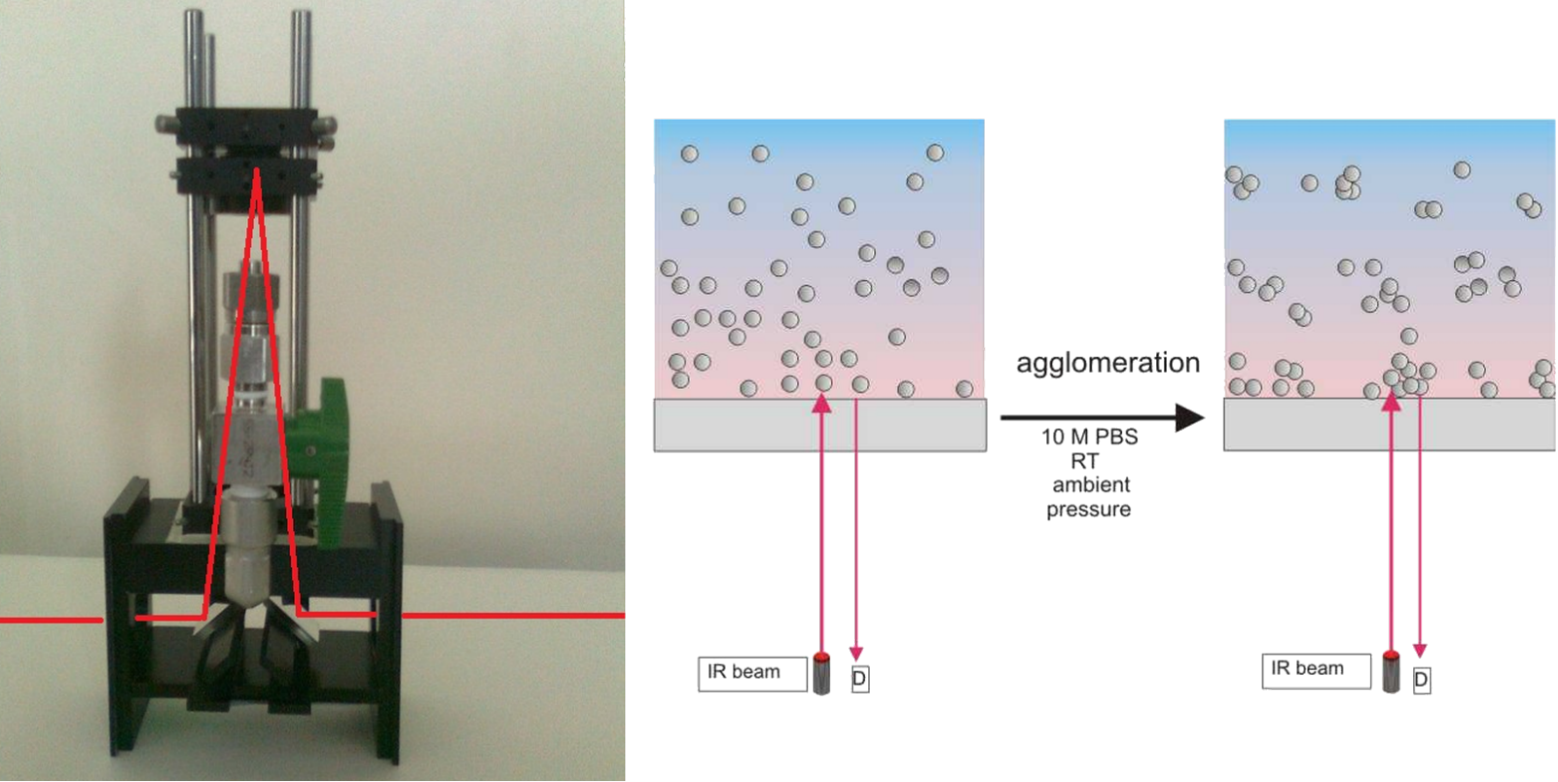
Photo of the RIfS Setup with inserted light path (left); RIfS transducer with nanoparticle solution (right).

Figure 4 shows the RIfS setup: the IR beam is reflected by a planar mirror to the 6 mm thick GaAs etalon of 25.4 mm diameter, coated with the polymer (either Teflon or PC), which acts as interference layer of the RIfS sensor. The reflection of the sensor is then guided to the MCT detector by reflection at a second planar mirror. A cell on top of the polymer holds the sample.

### Sensor chip preparation

The sensor was coated in one case with Teflon AF2400, in the other case with polycarbonate (PC). The Teflon AF2400 solution (2000 rpm at room temperature) was spin-coated onto the GaAs etalon in several layers (final layer thickness 1.65 µm, refractive index 1.29). Solid polycarbonate was solved in cyclopentanone (concentration 2.1 wt %) and spin-coated onto the GaAs etalon in a single step at 2000 rpm (layer thickness 0.78 µm, refractive index is 1.585). The layer thickness was calculated from the IR transmission spectrum using the Fabry–Pérot relationship 2*nd* = *m*λ, where λ represents the wavelength, *m* the interference order, *n* the refractive index of the polymer, and *d* the polymer thickness. The refractive index data were taken from the literature [23–24].

#### Modeling

During sensor design considerations some model calculations were performed. The reflectance response of multilayers was calculated using a MatLab toolbox developed by Orfanidis and described in his textbook “RF antenna theory” [25].

#### Materials

Common chemicals of analytical grade were purchased from Sigma-Aldrich (Deisenhofen, Germany) or Merck (Darmstadt, Germany). Teflon AF2400 was purchased from DuPont (USA). The nanoparticles used in the experiments were polyvinylpyrrolidone (PVP) stabilized spherical silver nanoparticles (AgNP) with a diameter of 51 nm in aqueous suspension, AgNP content 0.01% (w/w) and PVP content 0.2% (w/w). The nanoparticle suspension was used as delivered.
